# Host Restrictive Factors Are the Emerging Storm Troopers Against Enterovirus: A Mini-Review

**DOI:** 10.3389/fimmu.2022.910780

**Published:** 2022-05-04

**Authors:** Chen Huan, Xinglong Qu, Zhaolong Li

**Affiliations:** ^1^ Center of Infectious Diseases and Pathogen Biology, Institute of Virology and AIDS Research, Key Laboratory of Organ Regeneration and Transplantation of The Ministry of Education, The First Hospital of Jilin University, Changchun, China; ^2^ Respiratory Department of the First Hospital of Jilin University, Changchun, China

**Keywords:** host restrictive factors, enteroviruses, virus-host interplay, antiviral, arms race

## Abstract

Enterovirus infection continues to be a global health problem. The lack of specific drugs and broad-spectrum vaccines means an urgent need to develop effective strategies against enteroviruses. Host restrictive factors are a class of intrinsic host antiviral factors that have been broadly defined and investigated during HIV infections and have great significance for drug development and treatment design. In recent years, the essential role of host restrictive factors in regulating enteroviral infections has been gradually recognized and investigated. An increasing number of studies have shown that host-restrictive factors regulate multiple steps in the life cycle of enteroviruses. This mini-review discusses the restrictive factors against enteroviruses, their antiviral mechanism, and the arms race between them and enteroviruses. We also summarise the pathways that enteroviruses use to impair host antiviral signals. This mini-review characterizes the essential role of host restriction factors in enterovirus infections, which provides ideas and potential targets for antiviral drug design by regulating host restrictive factors. It also reveals potential future research on the interplay between host restrictive factors and enteroviruses.

## Introduction

There are more than 100 subtypes of enteroviruses that infect humans, including the well-known enterovirus 71 (EV71), enterovirus D68 (EVD68), coxsackieviruses A and B, and poliovirus (PV) (1), and several subtypes of these induce hand-foot-and-mouth disease (HFMD) epidemics every year (2–5). Moreover, EVD68 has been the cause of an unprecedented epidemic of respiratory disease, whose symptoms are unlike its common symptoms and have been temporally associated with acute flaccid myelitis (AFM) (6–8). However, the lack of effective drugs and broad-spectrum vaccines has exacerbated severe health problems.

A series of studies have investigated the interactions between host innate immunity and enteroviruses. Host restriction factors are expressed and/or induced in response to virus infection and include proteins from interferon-stimulated genes (ISGs) (9–19). APOBEC3G (A3G), SAMHD1, and BST2 have been extensively investigated in HIV infection (13). Since the antiviral effects of restrictive factors tend to have a broad spectrum, the regulatory function of host restriction factors during enterovirus infection has been investigated in recent years and has become a rising focus of enterovirus research. For example, we identified A3G and SAMHD1 restricted multiple enteroviruses and revealed a novel antiviral mechanism (20–23). IFNs and NF-κB signals are activated by viral infection (24–30), which induces the expression of downstream host-restrictive factors to fight against viruses *via* their pathways. However, viruses can impair restriction through multiple strategies. Here, we discuss the host restriction factors that play essential roles in regulating enteroviruses, the underlying mechanism they suppress, and how enteroviruses break host restrictions. This mini-review provides new information that can be used to select potential drug targets against enteroviruses and enlighten the direction of future studies in antivirus research.

## Host Restrictive Factors Play Antiviral Roles During Enteroviruses Infection

**Figure 1:**
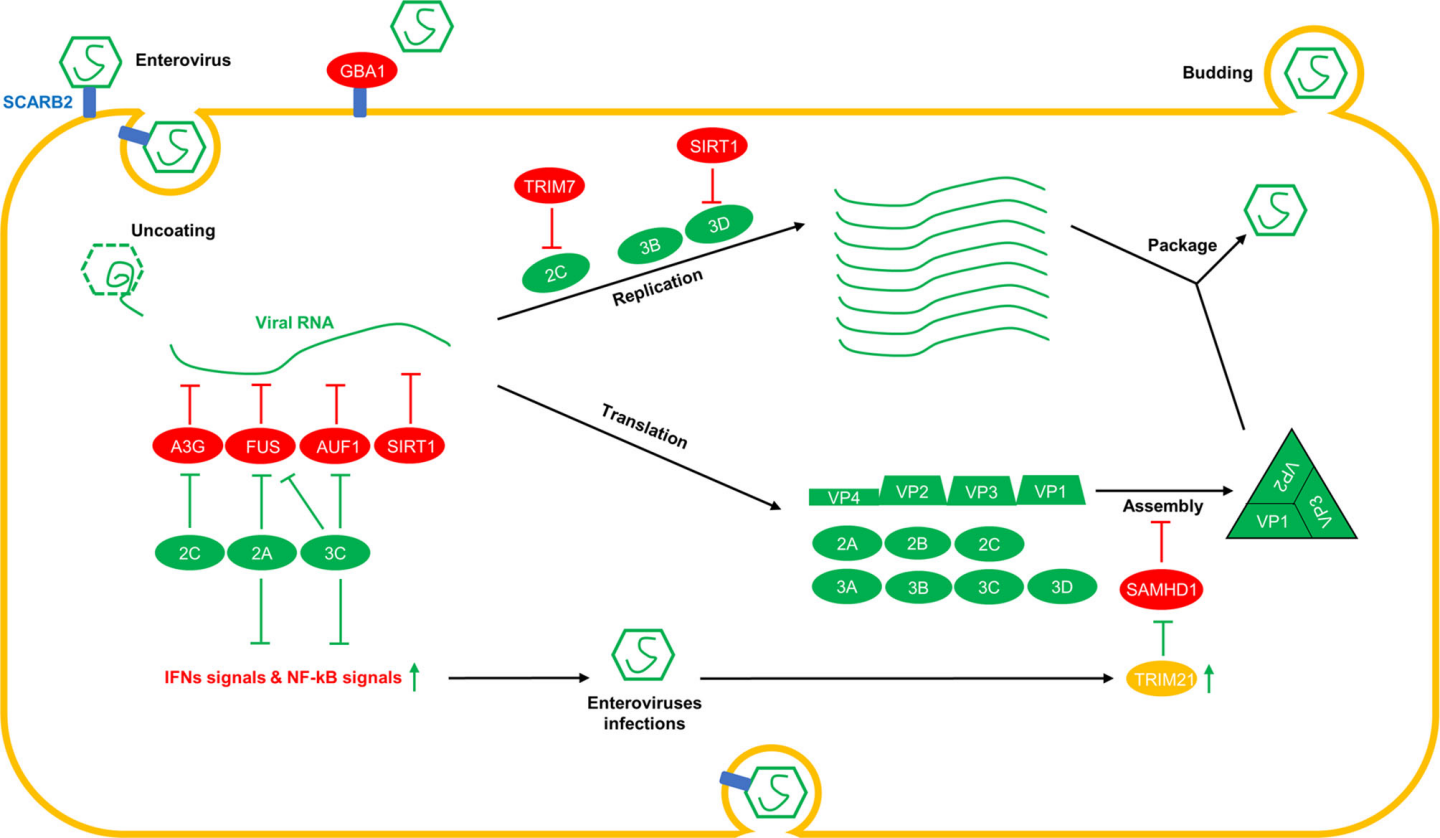
Overview of the interplay between host factors and enterovirus replication. Various host-restrictive factors have been shown to play regulatory roles at different stages of the virus life cycle. During the invasion phase, GBA1 interacts with SCARB2, a receptor of enteroviruses, and interferes with the binding of enteroviruses to SCARB2. After uncoating, A3G, FUS, AUF1, and SIRT1 interact with viral RNAs and reduce the replication and translation of viral RNAs. During the viral RNA replication phase, TRIM7 induces the degradation of 2C, and SIRT1 triggers the deacetylation of 3D^pol^, which is required for viral RNA replication. During the assembly phase, SAMHD1 interacts with VP1 and disrupts viral capsid assembly by interfering with the interactions between the viral capsid proteins VP1 and VP2. To break through the restriction from the host, viral proteins, such as 2A^pro^ and 3C^pro^, cleave FUS, other IFNs and NF-kB signal-associated proteins, and 3C^pro^ cleaves AUF1. Furthermore, 2C induces the degradation of A3G. TRIM21, which is upregulated by enteroviruses, triggers polyubiquitination and the degradation of SAMHD1.

Many in-depth studies on the interaction between enteroviruses and host factors have shown that host-restrictive factors play regulatory roles in different stages of the viral life cycle. We have summarised these studies based on the lifecycles of enterovirus infections (**Figure 1** and **Table 1**).

**Table 1:** Host restrictive factors identified in this review.

Gene names	Viral type	Antiviral mechanism	References
A3G	EV71, CA16, EVD68	1. Competitively binds to 5’UTR along with PCBP1. 2. Interacts with 3D^pol^ and is packaged into progeny virions.	(26, 31) (32)
AUF1	PV, CVB3	1. Interacts with viral IRES.	(33–35)
FUS/TLS	CVB3	1. Interacts with viral RNA. 2. Formation of stress granules and regulates innate immunity.	(36)
GBA1	EV71	1. Reduces the expression of SCARB2 on the cell surface. 2. Interferes with EV71 binding to SCARB2.	(17)
SAMHD1	EV71, CA16, EVD68	1. Interferes with the interactions between VP1 and VP2.	(37, 38)
SIRT1	EV71	1. Interacts with viral 5’UTR. 2. Reduces the acetylation of 3D^pol^.	(39)
TRIM7	EV71, CVB3, E11, EVD68, PV	1. Degrades viral 2BC protein.	(40)

Host restrictive factors are listed in alphabetical order.

## Viral Entry

Enteroviruses invade host cells by first interacting with specific receptors on the cell surface, where virions are endocytosed, or viral nucleic acids are released into the cells. For instance, scavenger receptor class B member 2 (SCARB2) was identified as a receptor for EV71 by Yamayoshi et al. (41). On this basis, Nakata et al. reported that acid beta-glucosidase 1 (GAB1) restricts EV71 infections by interacting with SCARB2 and reducing the expression of SCARB2 on the cell surface which interferes with the interactions between EV71 and SCARB2 (42). Further investigations showed that recombinant human GBA1, a molecular drug originally used to treat Gaucher’s disease (33, 40, 43), protected against EV71 infection (42), hinting that researchers could design anti-enterovirus drugs based on host restrictive factors.

## Viral RNA Replication and the Protein Translation Phases

After entering host cells, RNAs from enteroviruses are replicated and translated into viral proteins under the regulation of host factors (22, 34–36, 39, 44) and viral proteins, including 2BC, 3AB, and 3D (31, 32, 45–49). During this phase, many restrictive factors are involved in inhibiting virus replication. TRIM7, an E3 ligase, has been reported to restrict the replication of multiple enteroviruses by triggering polyubiquitination of their 2BC proteins and inducing the degradation of 2BC proteins *via* the proteasomal pathway (50).

AU-rich element degradation factor 1 (AUF1) binds to the internal ribosome entry site (IRES) of viral RNA and restricts the replication of poliovirus and CVB3 (51–53). However, the 3C^pro^ component of enteroviruses can cleave AUF1 to break through the restriction (53).

Like AUF1, fused sarcoma/translocated in liposarcoma (FUS/TLS) is a novel host antiviral factor that restricts CVB3 replication by directly inhibiting viral RNA transcription and protein translation. Moreover, FUS, which binds to viral RNA, triggers the formation of stress granules and regulates the activity of host antiviral innate immunity (54). CVB3 infection induces cytoplasmic mislocalization and cleavage of FUS through the enzymatic activity of viral proteases to evade the FUS-mediated antiviral response and innate immunity (54). In addition, as a class III NAD+-dependent histone, the deacetylase (HDAC), SIRT1, suppresses EV71 replication by repressing viral RNA transcription and attenuating viral RNA translation (55). Mechanistically, Han et al. identified the interactions between SIRT1 and viral 3D^pol^, and revealed that SIRT1 inhibits 3D^pol^ activity by reducing the acetylation of 3D^pol^. They also found that SIRT1 was able to interact with the viral 5’-UTR and interfere with viral RNA transcription and translation. Additionally, the expression of SIRT1 is upregulated by EV71 infection (55). However, Li et al. observed that EV71 infections could reduce the expression of SIRT1, and administration of the ROS inhibitor N-acetyl-L-cysteine (NAC) reduced apoptosis levels and inflammation, downregulated EV71 propagation, and increased SIRT1 expression in EV71-infected cells (56). Nevertheless, the mechanism underlying the regulation of SIRT1 expression induced by EV71 infection remains unclear.

A3G has been identified as a host-restrictive factor that suppresses HIV replication *via* cytosine deaminase activity (37, 38). In recent years, A3G has been shown to restrict the replication of enteroviruses, such as EV71, CA16, and EVD68, but not CA6 (20, 22, 57). These reports propose novel antiviral mechanisms independent of the cytosine deaminase activity shown by A3G. Li et al. suggested that while suppressing multiple enteroviruses, A3G competitively binds to the viral 5’UTR together with PCBP1, which is required for the transcription and translation of viral RNAs (22, 57). Further investigation showed that PCBP2, but not PCBP1, was required for CA6 replication, which would explain why A3G failed to restrict CA6 replication (22). In addition, Wang et al. suggested that A3G interacts with the 3D^pol^ of EV71 and packages it into progeny virions to reduce its infectivity (20). They also found that an inhibitor named IMB-Z inhibited EV71 replication by upregulating the expression of A3G (20). These studies confirmed that A3G inhibits enterovirus and that the mechanism was independent of its cytosine deaminase activity. In addition, other members of the APOBEC3 family, including A3A, A3D, and A3F, were found to possess antiviral activity against EV71 (57).

In contrast, Li et al. reported that A3G is degraded by the 2C proteins in enteroviruses, including EV71, CA6, CA16, CVB3, and EVD68 (57). In their study, viral 2C proteins triggered the polyubiquitination of A3G. Then the polyubiquitinated A3G was recognized by P62 and degraded by autolysosomes.

## Viral Assembly Phase

Like A3G, SAMHD1, another anti-HIV-restrictive factor, has been extensively investigated (14, 58, 59). SAMHD1 inhibits multiple retroviruses and DNA viruses (60–62), but its antiviral mechanism is unclear. Several studies have suggested that SAMHD1 restricts viruses through its dNTPase activity (14, 60, 63–65), and other studies have argued that its nuclease activity also contributes to its antiviral activity (66–68). For enteroviruses, Li et al. reported that SAMHD1 restricted EV71 replication independently of the dNTPase and nuclease activity of SAMHD1 (21). Furthermore, Zhao et al. reported that SAMHD1 restricted the replication of enteroviruses, including EV71, EVD68, and CA16, but not CA6, by interfering with the interactions between the viral capsid proteins VP1 and VP2 (23). Zhao et al. showed that SAMHD1 interacts with the EV71-VP1 domain, which is essential for the interaction between EV71-VP1 and EV71-VP2 and attenuates the interaction between EV71 VP1 and VP2. However, the interaction between SAMHD1 and CA6-VP1 did not disrupt the interaction between VP1 and VP2 of CA6, which may explain why SAMHD1 failed to inhibit CA6 (23).

In response to the inhibition of SAMHD1, EV71 has evolved a strategy to overcome this restriction and ensure the survival of its progeny. In a study by Li et al., EV71 infection induced proteasome-associated degradation of SAMHD1 by upregulating the expression of E3 ligase TRIM21, which triggers the polyubiquitination SAMHD1. TRIM21 upregulation is interferon receptor-dependent (21). Li et al. also identified the interaction domains between SAMHD1 and TRIM21 and the ubiquitination site on SAMHD1, which may provide clues for further drug target screening and design.

Several host restrictive factors play essential regulatory roles in various stages of the enterovirus life cycle, and exploring the mechanism underlying the interplay between host restrictive factors and enteroviruses will provide an important scientific basis for strategies against strategies for enterovirus infection.

## Enteroviruses Break Out of Host Restriction by Blocking Antiviral Pathways

During the long-term arms race, viruses evolve strategies to impair restriction from the host. In addition to the antagonistic strategies against the host restrictive factors mentioned above, enteroviruses also can disrupt other antiviral pathways to ensure their life cycle within the host (**Figure 1**). EV71 3C^pro^ has been reported to cleave multiple innate immune pathway-related proteins, including TRIF (26, 69), TRIM25 (70), TAK1, TAB1, TAB2, TAB3 (71), NLRP3 (72), IRF3 (73), IRF7 (74), IRF9 (75) and PMLIII and IV (76), and reduce IFN and NF-kB signals (77). As this type of research expands, the 3C^pro^ in EVD68 has also been reported to cleave IRF7 and affect IFN signaling (78, 79). Furthermore, the 2A^pro^ in EV71 has been reported to cleave MAVS, MDA5, and NLPR3 (72, 80, 81) and downregulate IFN and NF-kB signaling. In addition to these viral proteases, the 2C proteins in multiple enteroviruses have been reported to suppress NF-kB and IFN signals by binding to IKKβ, P65, and MDA5 (82–86). After that, reducing the antiviral signal levels will decrease the expression of antiviral factors, which contain many host restrictive factors and are beneficial to the unscrupulous replication of viruses.

## Discussion

Enterovirus infections are prevalent worldwide. However, the lack of specific drugs and broad-spectrum multivalent vaccines poses an urgent health threat. So, considerable studies on drug design targeting viral proteins have been conducted but unsuccessful (87). The high mutagenicity of RNA viruses and the similarity between the virus enzyme active domain and the host protein present considerable obstacles to selecting drug targets (88–92). The discovery of host restriction factors against enteroviruses and their interactions with viruses has attracted attention as a new antiviral strategy. Under this strategy, we could regulate the expression of host restrictive factors and effectively inhibit viral infections. Furthermore, we have identified the ‘Achilles heel’ of enteroviruses based on studies of hosting restrictive factors against enteroviruses. For instance, Zhao et al. reported that 119-223aa in VP1 were essential for the interactions between VP1 and VP2 (23). Based on this assumption, the inhibitors targeting 119-223aa in VP1 would possess a space-occupying effect and restrict the replication of enteroviruses, which may be a promising drug against EV71 infection.

On the other hand, viruses have evolved various methods to overcome the restriction of host restrictive factors, and treatment design against enteroviruses from this perspective will kill two birds with one stone. As reported by Li et al., the 2C protein of enteroviruses interacts with A3G and triggers the degradation of A3G (22, 57). Thus, inhibitors targeting the 2C domain that binds A3G can interfere with the interaction between the 2C protein and A3G and prevent the escape of the enteroviruses from A3G. At the same time, even if the target domain of the 2C protein mutates and causes the effects of inhibitors to be off-target, the mutant 2C protein will fail to bind to A3G and break out the restriction from A3G, indicating that A3G could exert its antiviral activity and that the inhibitors targeting this domain will stably inhibit enteroviruses by inducing virus mutation to a greater extent.

Third, as the endogenous component of host cells, it is important to note that antiviral strategies that regulate the expression of host restrictive factors will greatly reduce any side effects, which will be milder and safer than those experienced after using drugs. As Wang et al. showed in their study, IMB-Z inhibits EV71 replication by upregulating the expression of A3G (20). Moreover, 80 µM IMB-Z induced adequate A3G expression and greatly inhibited the replication of EV71 in a variety of cells. At the same time, 200 µM IMB-Z did not affect cell activity in cell lines, including Vero, HeLa, HCT-8, HEK293T, and SK-N-SH. Therefore, these findings have implications for the safety of antiviral strategies against enteroviruses by regulating the expression of host-restrictive factors.

In recent years, antimicrobial peptides (93) and mRNA drugs (94) have attracted increased interest among scientists, health professionals, and pharmaceutical companies because of their therapeutic potential. With the development of polypeptide and mRNA drugs, the functional domains of host restrictive factors will rapidly develop into antiviral drugs and become the mainstay of novel antiviral therapies. Therefore, identifying human host restrictive factors and exploring the interaction mechanism between a virus and host restrictive factors will become the premise and basis for us to master important antivirus strategies in the future.

In conclusion, studies on the interactions between host restrictive factors and enteroviruses will deepen understanding of virus-host interactions, provide a theoretical basis, and reveal potential targets that are not prone to off-target effects. This information can then be used to develop anti-enterovirus drugs.

## Author Contributions

ZL conceptualized the ideas. XQ performed the literature search, drafted the original manuscript, and drew the figures. CH revised the manuscript. All the authors approved the final version of the manuscript.

## Funding

This work was supported in part by funding from the National Natural Science Foundation of China (81701987 to ZL and 81801994 to CH), the Science and Technology Department of Jilin Province (20210101300JC), China Postdoctoral Science Foundation (2020M670826), and the Education Department of Jilin Province (JJKH20211141KJ).

## Conflict of Interest

The authors declare that the research was conducted in the absence of any commercial or financial relationships that could be construed as a potential conflict of interest.

## Publisher’s Note

All claims expressed in this article are solely those of the authors and do not necessarily represent those of their affiliated organizations, or those of the publisher, the editors and the reviewers. Any product that may be evaluated in this article, or claim that may be made by its manufacturer, is not guaranteed or endorsed by the publisher.
